# Identification of Candidate Genes for a Major Quantitative Disease Resistance Locus From Soybean PI 427105B for Resistance to *Phytophthora sojae*

**DOI:** 10.3389/fpls.2022.893652

**Published:** 2022-06-14

**Authors:** Stephanie Karhoff, Christian Vargas-Garcia, Sungwoo Lee, M. A. Rouf Mian, Michelle A. Graham, Anne E. Dorrance, Leah K. McHale

**Affiliations:** ^1^Center for Applied Plant Sciences, The Ohio State University, Columbus, OH, United States; ^2^Center for Soybean Research, The Ohio State University, Columbus, OH, United States; ^3^Department of Horticulture and Crop Science, The Ohio State University, Columbus, OH, United States; ^4^United States Department of Agriculture-Agricultural Research Service, Soybean Research Unit, Raleigh, NC, United States; ^5^Department of Agronomy, Iowa State University, Ames, IA, United States; ^6^United States Department of Agriculture-Agricultural Research Service, Corn Insects and Crop Genetics Resources Unit, Ames, IA, United States; ^7^Department of Plant Pathology, The Ohio State University, Wooster, OH, United States

**Keywords:** soybean, quantitative disease resistance, RNA-seq, *Phytophthora sojae*, jasmonic acid, serine-threonine kinase, *Glyma.18g026900*, glutathione

## Abstract

Phytophthora root and stem rot is a yield-limiting soybean disease caused by the soil-borne oomycete *Phytophthora sojae*. Although multiple quantitative disease resistance loci (QDRL) have been identified, most explain <10% of the phenotypic variation (PV). The major QDRL explaining up to 45% of the PV *were* previously identified on chromosome 18 and represent a valuable source of resistance for soybean breeding programs. Resistance alleles from plant introductions 427105B and 427106 significantly increase yield in disease-prone fields and result in no significant yield difference in fields with less to no disease pressure. In this study, high-resolution mapping reduced the QDRL interval to 3.1 cm, and RNA-seq analysis of near-isogenic lines (NILs) varying at *QDRL-18* pinpointed a single gene of interest which was downregulated in inoculated NILs carrying the resistant allele compared to inoculated NILs with the susceptible allele. This gene of interest putatively encodes a serine–threonine kinase (STK) related to the *AtCR4* family and may be acting as a susceptibility factor, based on the specific increase of jasmonic acid concentration in inoculated NILs. This work facilitates further functional analyses and marker-assisted breeding efforts by prioritizing candidate genes and narrowing the targeted region for introgression.

## Introduction

The United States produced 4.1 billion bushels of soybean (*Glycine max* (L.) Merr.) in 2020 (SoyStats, 2020). Yet, pests and pathogens continue to cause substantial yield loss each year. Phytophthora root and stem rot (PRR) is caused by the soil-borne oomycete *Phytophthora sojae* (Kaufmann and Gerdemann, 1958). This homothallic hemibiotroph infects soybean roots throughout the growing season *via* motile zoospores (Schmitthenner, 1985). PRR is primarily managed *via* genetic host resistance. Breeders have historically relied on single, dominant *Rps* genes, which are qualitatively inherited and typically isolate-specific, though there are exceptions (Wang W. et al., 2021). However, this widespread use of *Rps* genes in soybean cultivar development combined with the rapid pace of *P. sojae* evolution has caused a shift in pathogen virulence (Stewart et al., 2016). Moreover, *P. sojae* populations have continued to adapt to the previously deployed *Rps* genes throughout most of the North Central US (Dorrance et al., 2016). The second type of host resistance, referred to as partial resistance, is quantitatively inherited and considered race non-specific. Partial resistance to *P. sojae* has also been utilized in soybean cultivar development and is conferred by multiple quantitative disease resistance loci (QDRL), although the majority of loci explain <10% of the phenotypic variation (PV) (Burnham et al., 2003; Weng et al., 2007; Han et al., 2008; Li et al., 2010; Tucker et al., 2010; Wang et al., 2010; Wu et al., 2011; Lee et al., 2014; Abeysekara et al., 2016; Schneider et al., 2016; Stasko et al., 2016; Rolling et al., 2020).

While rare in the soybean *P. sojae* pathosystem, three major QDRL for partial resistance to *P. sojae* have been previously reported (Tucker et al., 2010; Lee et al., 2014; de Ronne et al., 2021). Among those, the major QDRL referred to as *QDRL-18* have been identified on chromosome 18 (981-2,833 kb) (Lee et al., 2014; Karhoff et al., 2019), distal (>48.1 Mbp) from the six *Rps* genes previously mapped to this chromosome (Diers et al., 1992; Demirbas et al., 2001; Sandhu et al., 2004; Sun et al., 2014; Sahoo et al., 2017, 2021). *QDRL-18* was mapped in two separate recombinant inbred line (RIL) populations, with resistance alleles from plant introduction (PI) 427106 and PI 427105B and explained up to 45% of the PV (Lee et al., 2014). Studies utilizing near-isogenic lines (NILs) with resistant introgressions from PI 427106 and PI 427105B showed that the resistance alleles increased yield by 13–29% under field conditions that were highly favorable to PRR and significantly increased partial resistance in both laboratory and greenhouse assays (Karhoff et al., 2019). While the effect of *QDRL-18* on overall yield in the absence of disease pressure remains to be tested, *QDRL-18* represents a valuable source of partial resistance to *P. sojae* (Karhoff et al., 2019). The locus spans a genomic region of 1,852 kb, which includes 222 predicted genes based on the Williams 82 reference genome (Wm82.a2.v1; Schmutz et al., 2010).

Quantitative trait loci (QTL) often encompass hundreds of genes, making it difficult to determine the causal gene(s) underlying the QTL (St.Clair, 2010). Recent studies in cowpea (*Vigna unguiculata (L.))* (Santos et al., 2018), cotton (*Gossypium spp.)* (Li et al., 2017), wild rice (*Oryza rufipogon)* (Wang et al., 2017), soybean (McCabe et al., 2018; Jiang et al., 2021), wheat (*Triticum aestivum)* (Wang Y. et al., 2021), and common bean (*Phaseolus vulgaris L)* (Yang et al., 2022) have successfully identified the candidate genes for root-knot nematode resistance, fiber length, salt tolerance, brown stem rot resistance, cyst nematode, stripe rust, and bacterial blight, respectively, by coupling linkage mapping with RNA-seq expression analyses. Thus, the integration of mapping and gene expression information may be a promising method for characterizing complex loci such as *QDRL-18*.

In this study, we evaluated the effect of *QDRL-18* on yield in low disease environments, combined gene expression analyses (RNA-seq) and high-resolution QTL mapping to identify candidate genes associated with *QDRL-18*, and quantified salicylic acid and jasmonic acid in inoculated and mock-inoculated NILs to reveal potential mechanisms by which *QDRL-18* functions. Overall, this work will facilitate the introgression of *QDRL-18* and future functional analyses of candidate genes to elucidate the mode of action and potential pathways involved.

## Materials and Methods

### Plant Material

A total of two F_7:8_ RIL populations originally developed by Lee et al. (2014) were used in this study. The first population, “OX20-8” × PI 427106 (OP3), contained 367 individuals, and the second, OX20-8 × PI 427105B (OP4), included 338 individuals. OX20-8 was developed in Ontario, Canada and is highly susceptible to *P. sojae* with the *Rps1a* gene and low level of partial resistance (Buzzell, 1982; Mideros et al., 2007). In contrast, PI 427105B and PI 427106 originate from the Jilin province in China and have high levels of partial resistance to *P. sojae* (Dorrance and Schmitthenner, 2000).

Near-isogenic lines were developed from RIL 4213, derived from the crosses between OX20-8 and PI 427105B, as previously described in Karhoff et al. (2019). Briefly, RIL 4213 was selected for heterozygosity at three single-nucleotide polymorphisms (SNPs) flanking and within *QDRL-18*: BARC-020839-03962, BARC-025777-05064, and BARC-047665-10370. Homozygous single plants derived from self-pollination of RIL 4213 were selected based on the two simple sequence repeats (SSRs) within the region of interest (BARCSOYSSR_18_0129 and BARCSOYSSR_18_0164) and advanced two generations to develop F_8:10_ NILs. In this study, five NILs with the resistant introgression and phenotype (average lesion length of 18.17 mm) and five NILs with the susceptible introgression and phenotype (average lesion length of 27.29 mm) were used (Karhoff et al., 2019). Parental lines OX20-8 and PI 427105B were also included as controls and continuously showed the phenotypic contrast.

### Genotyping, Genetic Map Reconstruction, and QDRL Analysis

The RIL populations were previously genotyped with 230 and 221 SNP and SSR markers polymorphically between OX20-8 and PI 427106 and OX20-8 and PI 427105B, respectively (Lee et al., 2014). In this study, RILs were genotyped with an additional nine SNP markers spanning the QDRL-18 target region (Supplementary Table S1). Polymorphic nucleotides were selected from the SoySNP50K SoyBase database (Song et al., 2013, 2015) and assays developed for the Kompetitive allele-specific PCR (KASP) platform (He et al., 2014; Semagn et al., 2014). KASP assays were performed in Bio-Rad Multiplate™ 96-well reaction plates (Bio-Rad Laboratories) on an Eppendorf Mastercycler pro S (Eppendorf). A 10 μl reaction volume consisting of 5 μl DNA (5–50 ng) and 5 μl 2x KASP master mix (LGC Genomics) was used. Thermal cycling conditions were as follows: 94°C for 15 min, followed by ten touchdown cycles at 94°C for 20 s and 61°C for 1 min (dropping 0.6°C per cycle), and 29 cycles at 94°C for 20 s and 55°C for 1 min. Bio-Rad CFX Manager software version 3.1 (Bio-Rad Laboratories) was used for SNP genotype calling.

The additional nine KASP markers were added to the genotypic data from Lee et al. (2014), for a total of 233 and 224 genetic markers on the populations derived from crosses between OX20-8 and PI 427106 and between OX20-8 and PI 427105B, respectively. A new genetic map was constructed for each of the individual populations with Kosambi mapping function in JoinMap 4.0 (Van Ooijen, 2006). The maximum likelihood mapping algorithm was used with a logarithm of odd (LOD) threshold of 3 for grouping. Composite interval mapping (CIM) was performed in MapQTL 5 (Van Ooijen, 2004) using the original best linear unbiased predictor (BLUP) values described by Lee et al. (2014). Genome-wide LOD thresholds were calculated using permutation tests with 1,000 iterations.

### Field Evaluation

Yield trials were conducted in two contrasting environments: those with a history of frequent flooding and severe PRR and those with reduced or less PRR history. The four trials with disease conducive environments were located in Defiance County (2015), Van Wert County (2016–2017), and Wood County (2021), Ohio, with data from the first three trials previously reported (Karhoff et al., 2019). The four environments with reduced disease pressure from *P*. *sojae* were in Wayne County (2019–2021) and Wood County, Ohio (2021). All field trials followed the split-plot design described by Karhoff et al. (2019) with the main plot corresponding to the NIL family and the subplot representing each line. Parental lines were included in field trials as checks but were removed from the final analysis. The effect of QDRL-18 on yield under each environment type (conducive or not conducive to PRR) was tested independently with analysis of variance (ANOVA) in R version 3.6.3 (R Core Team., 2018) using the package “lmerTest” (Kuznetsova et al., 2017). The linear mixed-effect model was utilized to test the effect of QDRL-18 which was Y= μ + (1| BE) + (1|E) + F + I(F) + ε where μ is the overall mean yield, B(E) is the random effect of the block nested within environment, E is the random effect of environment, F is the effect of the NIL set family, I(F) is the effect of introgression nested within NIL set family, and ε is the overall experimental error.

### *P. Sojae* Inoculation and Tissue Collection for RNA-Seq

Near-isogenic lines and parental lines were inoculated with *P. sojae* isolate 1.S.1.1 (*vir 1a, 1b, 1k, 2, 3a, 3b, 3c, 4, 5, 6, 7, 8*) in a tray test (Dorrance et al., 2008). Briefly, ten 7-day-old seedlings per line were placed on top of a 3-cm paper strip with the top 2 cm of the root on the paper strip and covered by a thick and a thin cotton/polyester cloth to retain moisture. Seedlings were inoculated 2 cm below the root crown with prepared zoospore suspension and diluted in sterile distilled water with an adjusted pH of 7.0, at a concentration of 1 × 10^4^ zoospores per ml (Mideros et al., 2007). Following inoculation, 1 cm of root tissue was collected at the inoculation site from 10 plants per line at 3, 24, and 48 h after inoculation (hai) and flash frozen in liquid nitrogen. A mock inoculation treatment consisting of sterile distilled water with an adjusted pH of 7.0 was also included for each line at each time point. The experimental design was a randomized complete block with three biological replications separated by time; one biological replication consisted of 12 trays, each containing ten plants per NIL or parental line for each treatment (inoculated and mock). To validate inoculation success, a set of 10 seedlings per line was maintained for 7 days, and lesion length was measured. Average lesion length between resistant and susceptible NILs was compared with a Welch's *t*-test using R version 3.5.0 “stats” package (R Core Team., 2018).

### RNA Isolation and Library Preparation

Total RNAs were isolated with Macherey-Nagel NucleoSpin^®^ RNA Mini Kit (Macherey-Nagel, Germany) according to the manufacturer's instructions. In total, there were 180 NIL samples (10 lines × 3 biological replications × 3 time points × 2 treatments) and 24 samples for parental lines OX20-8 and PI 427105B (2 lines × 2 biological replications × 3 time points × 2 treatments). An in-solution DNase digestion was performed per the manufacturer's protocol to remove DNA contamination in the RNA samples. The quality and quantity of RNA extracts were determined with Agilent 2100 Bioanalyzer™ (Agilent, United States) and Qubit fluorometer, and the minimum RNA integrity number of 8 was required. Library preparation and single-end sequencing were completed at the Iowa State University DNA facility with the Illumina HiSeq 2500 platform (Illumina, United States).

### Read Alignment and Illumina Sequence Analysis

Adapter sequences (Scythe, https://github.com/vsbuffalo/scythe), sequencing artifacts (FASTX trimmer, http://hannonlab.cshl.edu_fastx_toolkit), and low-quality bases (Sickle, https://github.com/najoshi/sickle) were trimmed, and read quality was confirmed (FastQC, https://github.com/s-andrews/FastQC). Reads were aligned to the assembly against Wm82.a2.v1 (Schmutz et al., 2010) with TopHat2 version 2.1.0 (https://github.com/infphilo/tophat) and counted with HTSeq (Anders et al., 2015). Genes with log2 counts per million (cpm) <1 in two or more replicates were removed, and remaining data for 33,873 genes were normalized using the Trimmed Mean of M (TMM) values (Robinson and Oshlack, 2010) in Bioconductor package edgeR (Robinson et al., 2009; McCarthy et al., 2012). Differentially expressed genes (DEGs) in (1) introgression-specific (resistant NILs vs. susceptible NILs) and (2) non-introgression-specific (inoculated vs. mock-treated) responses to *P. sojae* inoculation were identified at each time point in edgeR with a false discovery rate (FDR) of 0.05 and logFC threshold of 2.0. Chromosomal distribution of DEGs was evaluated with Fisher's exact test. Gene Ontology and Kyoto Encyclopedia of Genes and Genomes (KEGG) pathway enrichment analyses were performed for DEGs within each of the four comparisons at each time point with agriGO v2.0 (Tian et al., 2017), the database for annotation, visualization, and integrated discovery (DAVID) bioinformatics Resources v6.8. (Huang et al., 2009a,b), and R package “gage” (Luo et al., 2009), respectively. Pathway analysis results were visualized with R package “pathview” (Luo and Brouwer, 2013). Gene Ontology and enrichment analysis using DAVID evaluated DEGs identified between inoculated resistant vs. susceptible NIL introgression at all three points. Ranking of DEGs was based on functional categories of co-occurrences. Fisher's exact test was also utilized to measure gene enrichment within DAVID assigned clusters, DEGs biological grouping, and independent gene sets (α = 0.05) (Huang et al., 2009a,b).

### RT-qPCR of *Glyma.18g026900*

RNA-seq results were validated with RT-qPCR for *Glyma.18g026900* transcripts. Primers were designed with Primer3 software (http://bioinfo.ut.ee/primer3-0.4.0/; Koressaar and Remm, 2007). A total of 500 ng of RNA was reverse-transcribed with Bio-Rad iScript™ cDNA Synthesis Kit (Bio-Rad Laboratories, Hercules, California). Real-time quantification was performed in a CFX96 Touch™ Real-Time PCR Detection System (Bio-Rad Laboratories, Hercules, California) with the Bio-Rad iQ™ SYBR Green Supermix Kit. A 10 μl reaction volume containing 500 nM sense and anti-sense primers, 5 μl 1X SYBR Green PCR Master mix (Applied Biosystems), and 3 μl10x diluted cDNA was used. Thermal cycling conditions consisted of 3 min at 95°C, followed by 40 cycles of 10 s at 95°C, and 30 s at 56°C, ending with a melt curve analysis to verify amplification specificity. Standard curves were constructed with serial dilutions of cDNA run in triplicate, and primer efficiency was calculated (Pfaffl, 2004). A total of two technical replications were completed for each sample, and quantification cycle (Cq) values were averaged prior to data analysis. Gene expression was normalized to a constitutively expressed gene for roots identified by Libault et al. (2008), which putatively encodes an F-box protein. Relative expression of inoculated resistant NILs to inoculated susceptible NILs was calculated with the delta-delta Cq method (Livak and Schmittgen, 2001).

### *De novo* Transcriptome Assembly

Seqtk (https://github.com/lh3/seqtk) was used to randomly subsample 3 million reads per 180 NIL samples for a total of 540 million reads. A *de novo* assembly of these transcripts was created with Trinity version 2.8.4 (Grabherr et al., 2011). To ensure that 3 million reads were an appropriate level of subsampling, *de novo* transcriptomes were also assembled with 375,000, 750,000, and 1.5 million reads per NIL sample. The assembly quality was assessed by comparing the number of predicted soybean transcripts (Wm82.a2.v1) represented by nearly full-length transcripts having >80% alignment coverage (Supplementary Figure S1). The number of nearly full-length transcripts began to plateau at 3 million reads, and this sampling level was used to reduce computation load. Differentially expressed Trinity “genes” were identified by aligning reads to the *de novo* assembly with Bowtie 2 (Langmead and Salzberg, 2012), and transcript abundance was estimated with RSEM version 1.3.1 (Li and Dewey, 2011). The function was automatically assigned with Trinotate version 3.1.1 (Bryant et al., 2017), and differential gene expression analysis of count matrices was completed in EdgeR (Robinson et al., 2009) as described previously. Both Trinotate 3.1.1 and GOseq version 1.34.1 (Young et al., 2010) were used to conduct Gene Ontology enrichment of DEGs. Trinity “genes” were compared to predicted soybean transcripts (Wm82.a2.v1) with BLASTN (Altschul et al., 1990), and Trinity “genes” with no alignment were considered novel (E-value <1 × 10–20). Novel Trinity “genes” were compared with predicted soybean proteins (Wm82.a2.v1) with BLASTX (E-value <1 × 10–3; Altschul et al., 1990) to predict the function.

### *Glyma.18g026900* Sequence Analysis

Amino acid alignment of *Glyma.18G026900*, the Arabidopsis homolog and paralogs AtCCR3, AtCCR4, and AtCR4, and paralogs from soybean (Wm82.a2.v1) were built with Clustal Omega (Sievers and Higgins, 2014) using default options for parameters, and a neighbor-joining tree was built with Simple Phylogeny (Goujon et al., 2010) using distance correction and default parameters. A phylogeny was built using candidate gene *Glyma.18G026900* and was amplified from NILs set 4213-1 and 4213-32, which carried the susceptible (OX) and resistant (105B) introgression, and the parents, OX20-8 and PI 427105B. The DNA isolation was conducted using the DNeasy Plant Mini Kit (Qiagen, Hilden, Germany). A total of ten primer sets were constructed on Primer3 version 0.4.0 for traditional polymerase chain reaction (Koressaar and Remm, 2007; Untergasser et al., 2012). Each primer set spanned one-third of the sequence with a ~200-bp overlap. Upstream and downstream primer sets encompass ~1 kb upstream and downstream of *Glyma.18G026900*. PCRs were conducted using 2.5 ul 10X Standard Taq Reaction Buffer, 0.5 ul 10 mM dNTPs, 0.5 ul 10 μM Forward Primer, 0.5 ul 10 μM Reverse Primer, 2 ul DNA Template, 0.125 ul Taq DNA Polymerase, and 19 ul Nuclease-free water from New England Biolabs. The following conditions were used in an Eppendorf thermocycler: 95°C for 3 min, followed by 38 cycles of 95°C for 30 s, and 55–58°C for 30 s to finish with two rounds of 68°C for 30 s and 5 min, respectively. PCR amplicons were cleaned using “ExoSAP-IT™” (Applied Biosystems, Waltham, Massachuetts, USA) PCR reagent. Resulting amplicons were submitted to OSU-Biomedical Research Tower for Sanger Sequencing. Final sequences were cleaned using Benchling (Benchling [Biology Software], 2021). Contig assembly, sequence alignment, open reading frame (ORF) prediction, and the final sequence translation were done in Sequencher version 5.4.6. Protein modeling to identify conserved regions, key amino acids, and PROFbval catalytic activity was done in Phyre2/EzMol and Predict My Protein software (Kelley et al., 2015; Bernhofer et al., 2021). Final primers, sequences, and alignments can be found on a public GitHub repository (https://github.com/vargas-garcia/Glyma.18G026900). A one-way test of positive selection (Z-test) was performed in MEGA11 (Tamura et al., 2021) to evaluate the null hypothesis of dN = dS relative to the alternative hypothesis of positive selection (dN > dS), where dN is the number of nonsynonymous substitutions per nonsynonymous site and dS is synonymous substitutions per synonymous site.

### Salicylic (SA) and Jasmonic Acid (JA) Concentration Analysis

A second tray test using a subset of the NILs in similar experimental design as described above for “*P. sojae* Inoculation and Tissue Collection for RNA-Seq.” was done. In the earlier experiments, *Glyma.18G026900* had the greatest downregulation in resistant NILs at 24 hai. Thus, we used this time point and collected 36 samples [(2 Res NILs + 2 Sus NILs + 2 parents) × 2 treatments × 3 reps], for quantification of SA and JA. The data collected at 7 days after inoculation (dai) for lesion length assay confirmed the differential allele response between PI 427105B (resistant) and OX20-8 (susceptible) NILs (Supplementary Figure S2). Collected root crown samples were flash frozen in liquid nitrogen and then ground using a mortar and pestle until a fine powder was achieved. Before thawing, 0.8 mg of root tissue was collected and stored at −80°C. UPLC-MS-QqQ quantification of SA and JA was done by the Flavor Research and Education Center (FREC) services (https://frec.osu.edu/services). In brief, the powdered sample was mixed with 1 ml extraction solvent (90% methanol and 0.1% formic acid in water). A total of 10 μl of 1 μg/ml methylparaben (MP) was added to each sample as an internal standard. The mixture was homogenized in a Geno/Grinder at 1,000 rpm for 5 min followed by centrifugation at 12,000 × G for 5 min. The samples were then filtered through a PTFE filter (WAT200506, WATERS LC United States, 13 mm, 0.2 μm). About 2 μl of each sample was injected for LC-MS/MS analysis. Once in LC-MS/MS, compounds of interest were separated in WATERS LC system by a C18 column (2.1 x 100 mm, EC-C18 2.7 μm, Agilent poroshell 120) with water (containing 0.1% formic acid) as solvent A and acetonitrile (containing 0.1 % formic acid) as solvent B. The flow was set at 0.5 ml/min. The ramp started at 5% solvent B for 0.5 min; the solvent B was then increased to 60% in 4.5 min and 95% in 1 min; it was then held at 95% for 2 min and reduced to 5% in 0.1 min. The separated compounds were then analyzed and detected by MS/MS method and reported as ng/g. Only the first and second replications were considered in the final analysis due to the high uniformity achieved during zoospores inoculation, 8.0 × 10^4^ and 8.5 × 10^4^ zoospores/ml, respectively.

## Results

### QDRL Analysis

Using the previously generated genotypic data (Lee et al., 2014) as well as an additional nine SNPs spanning the *QDRL-18* interval, linkage maps were generated for the RIL populations OX20-8 (susceptible) x PI 427106 and OX20-8 x PI 427105B. These maps contained 233 and 224 markers distributed across 33 and 32 linkage groups (LGs), respectively. The OX20-8 x PI 427106 genetic map covered a total genetic distance of 2,212.9 cm with an average marker distance of 11.1 cm. The OX20-8 x PI 427105B genetic map covered a total genetic distance of 1,928.9 cm with an average marker distance of 10.1 cm.

**Figure 1:**
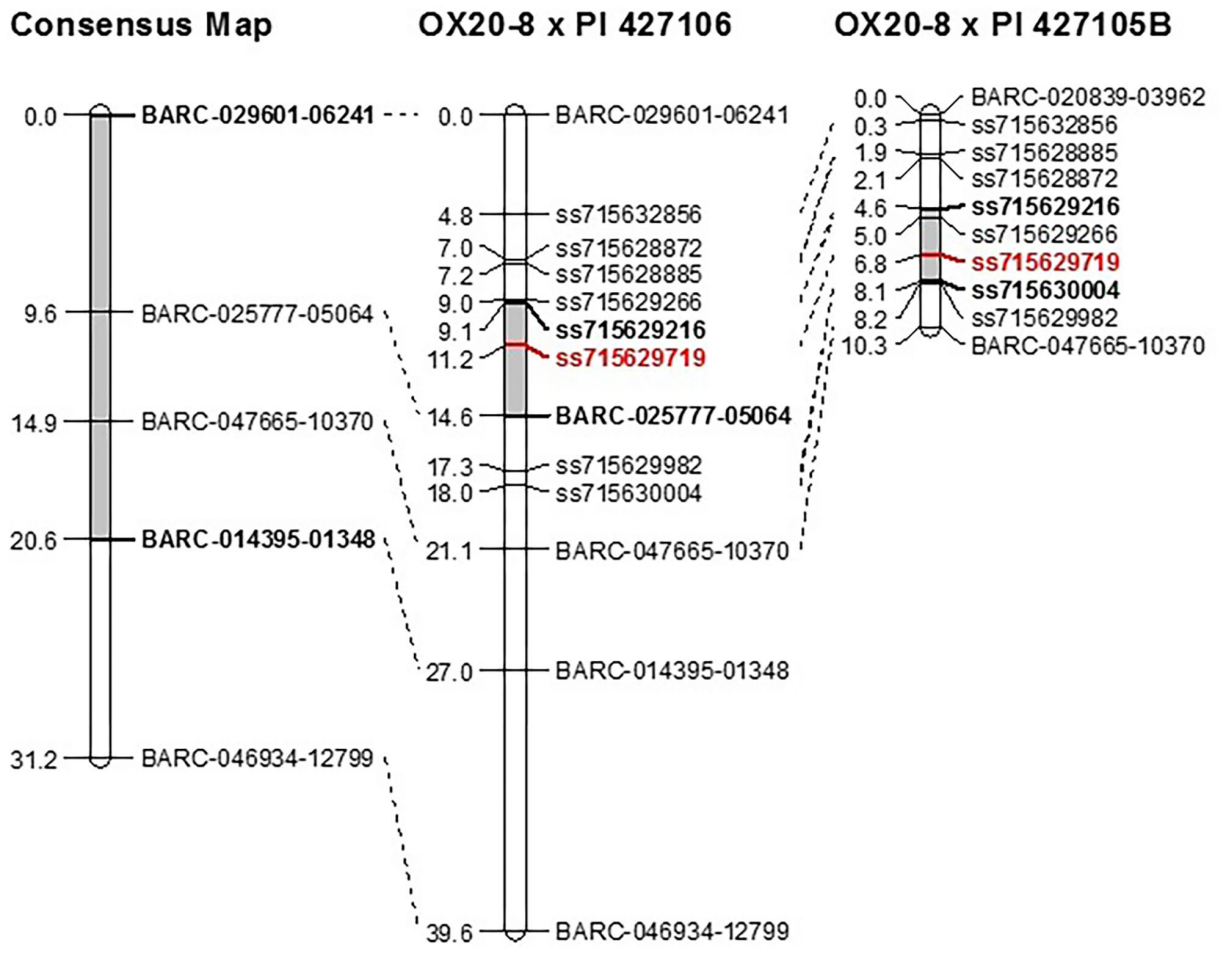
Genetic location of *QDRL-18* (shaded in gray) in Lee et al. (2014) consensus map compared to recombinant inbred populations OX20-8 x PI 427106 and OX20-8 x PI 427105B following addition of nine KASP markers. *QDRL-18* peak in this study (ss715629719) is in red. Figure was created using MapChart version 2.2 (Voorrips, 2002).

Composite interval mapping was performed in the two RIL populations to further delimit the genetic interval of *QDRL-18* (Table 1). In the OX20-8 x PI 427106 RIL population, *QDRL-18* explained 37% of PV and mapped to LG 18a, peaking at 11.20 cm, flanked by markers ss715629216 and BARC-025777-05064, which spanned a region of 3.1 cm (10.1–13.2 cm) (Figure 1). A minor QDRL explaining 7.8% of PV was mapped to LG 12 at 69.3 cm, with OX20-8 contributing the resistance allele. In the OX20-8 x PI 427105B RIL population, *QDRL-18* explained 24.2% of PV and mapped to LG 18a at position 6 cm, flanked by the same marker ss715629216 and by ss715630004 (4.1–8.1 cm) (Figure 1). Additionally, a minor QDRL explaining 5.1% of PV was mapped to LG 17 at 80.7 cm, with PI 427105B contributing the resistance allele.

**Table 1:** Quantitative disease resistance loci (QDRL) analysis for partial resistance to *Phytophthora sojae* isolate 1.S.1.1 in OX20-8 x PI 427106 and OX20-8 x PI 427105B recombinant inbred populations.

**PI source**	**LG**	**Pos (cm)**	**LOD**	**Thr^**†**^**	**PVE^**‡**^**	**Nearest marker**	**Left marker**	**Right marker**
PI 427106	12^§^	48-86	6.4	3.1	8%	BARC-061985-17608	BARC-019775-04370	BARC-044073-08598
PI 427106	18a	10-13	36.8	3.1	37%	ss715629719	ss715629216	BARC-025777-05064
*PI 427105B*	*17*	*70-105*	*3.3*	*3.0*	*5%*	*BARC-042295-08238*	*BARC-013637-01186*	*BARC-011591-00299*
PI 427105B	18a	4-8	19.8	3.0	24%	ss715629719	ss715629216	ss715630004

† *LOD threshold determined by a 1,000-permutation test*.

‡ *Phenotypic variance (%) explained by a single QDRL*.

§ *Italicized QDRL was not previously identified in Lee et al. (2014)*.

As determined by Lee et al. (2014), the original interval was 1,852 kb in size and encompassed 222 predicted soybean genes based on Wm82.a2.v1. Re-mapping of the *QDRL-18* locus narrowed the genomic interval by 40%, and the original *QDRL-18* interval (981–2,833 kb; Lee et al., 2014) was reduced to a 731-kb region (1,713 – 2,445 kb) containing 82 predicted genes based on Wm82.a2.v1 (Supplementary Table S2).

### Effect of *QDRL-18* on Yield Under High/Reduced Disease Conditions

**Figure 2:**
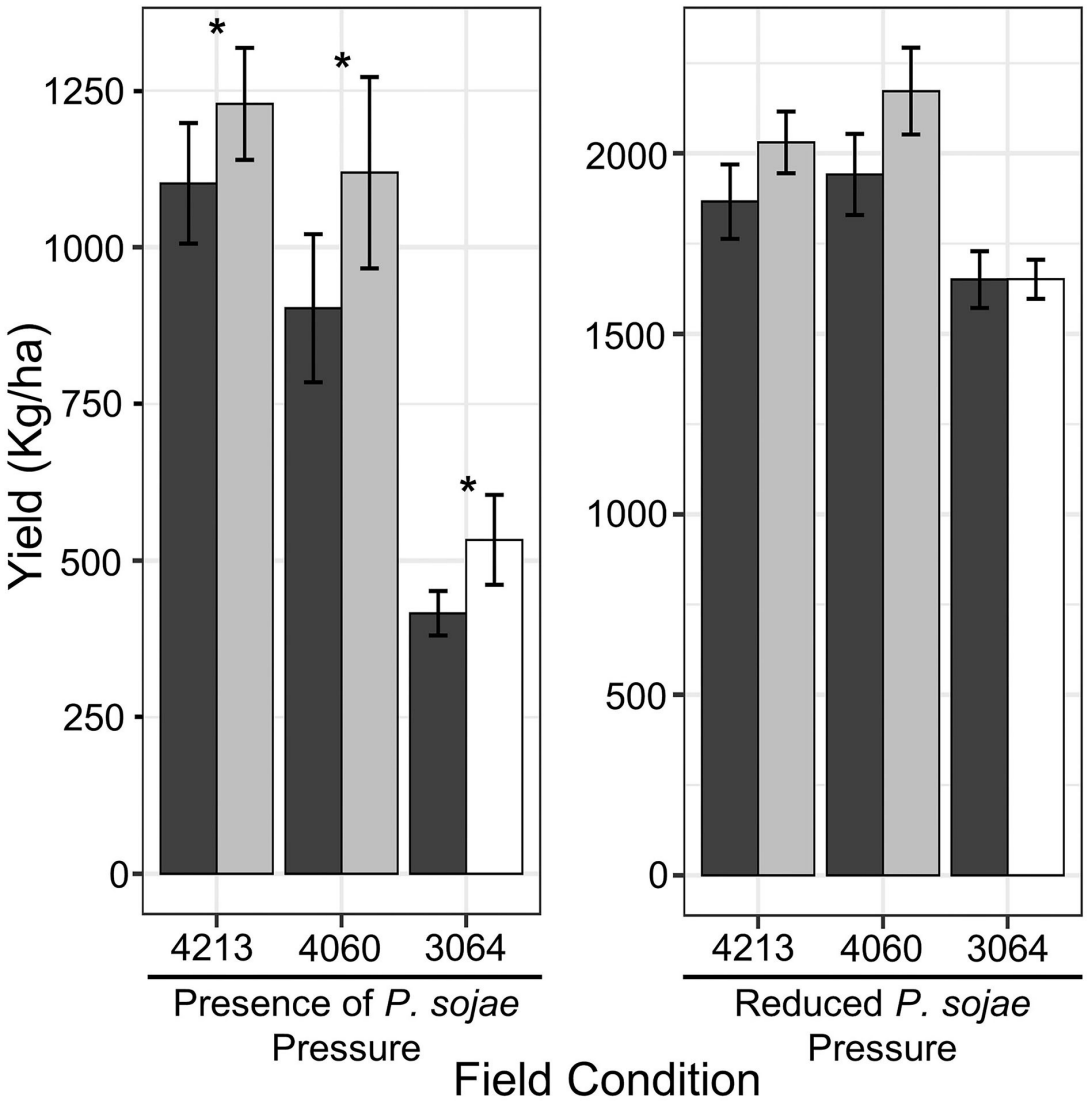
Field trial for yield stability of NILs families across a multiyear (2015–2017 and 2019–2021) experiment considering four and three location/environments with and without disease pressure respectively. Colors black (OX20-8), gray (PI 427105B), and white (PI 427106) illustrate NILs carrying the respective introgression. Error bars are represented by the standard error (±SE) whereas asterisk above the bars denote significance (*p < 0.05, Fisher's protected *t*-test).

While earlier studies had shown that the resistance allele could significantly increase yield under conditions conducive to PRR, we had not evaluated the effect of *QDRL-18* under field conditions which were not conducive to PRR. Therefore, the yield performance of NILs was tested in multiyear experiments, adding to the previous data, to evaluate the effect of *QDRL-18* on yield both in the presence and absence of environmental conditions conducive to this disease. In fields with a history of soil saturation and PRR, PRR was consistently observed on the susceptible OX20-8 (Karhoff et al., 2019). NILs carrying the resistant introgression significantly outperformed the susceptible introgression in all families with an average yield increase of 12, 24, and 28% in NIL family 4213, 4060, and 3064, respectively (α = 0.05, Fisher's protected *t*-test, Figure 2; *p* = 0.002 for allele within family, ANOVA; Supplementary Table S3). In contrast and in keeping with a function specific to resistance, there was no significant effect of *QDRL-18* on yield when NILs were grown in fields without a significant history of soil saturation or PRR (*p* > 0.05 for allele within family, ANOVA; Supplementary Table S3). Yet, in fields with no history or rare incidence of PRR, lines carrying the resistant introgression yielded numerically, slightly more than the susceptible introgression in each NIL set (Figure 2).

### Analysis of Differentially Expressed Genes

**Figure 3:**
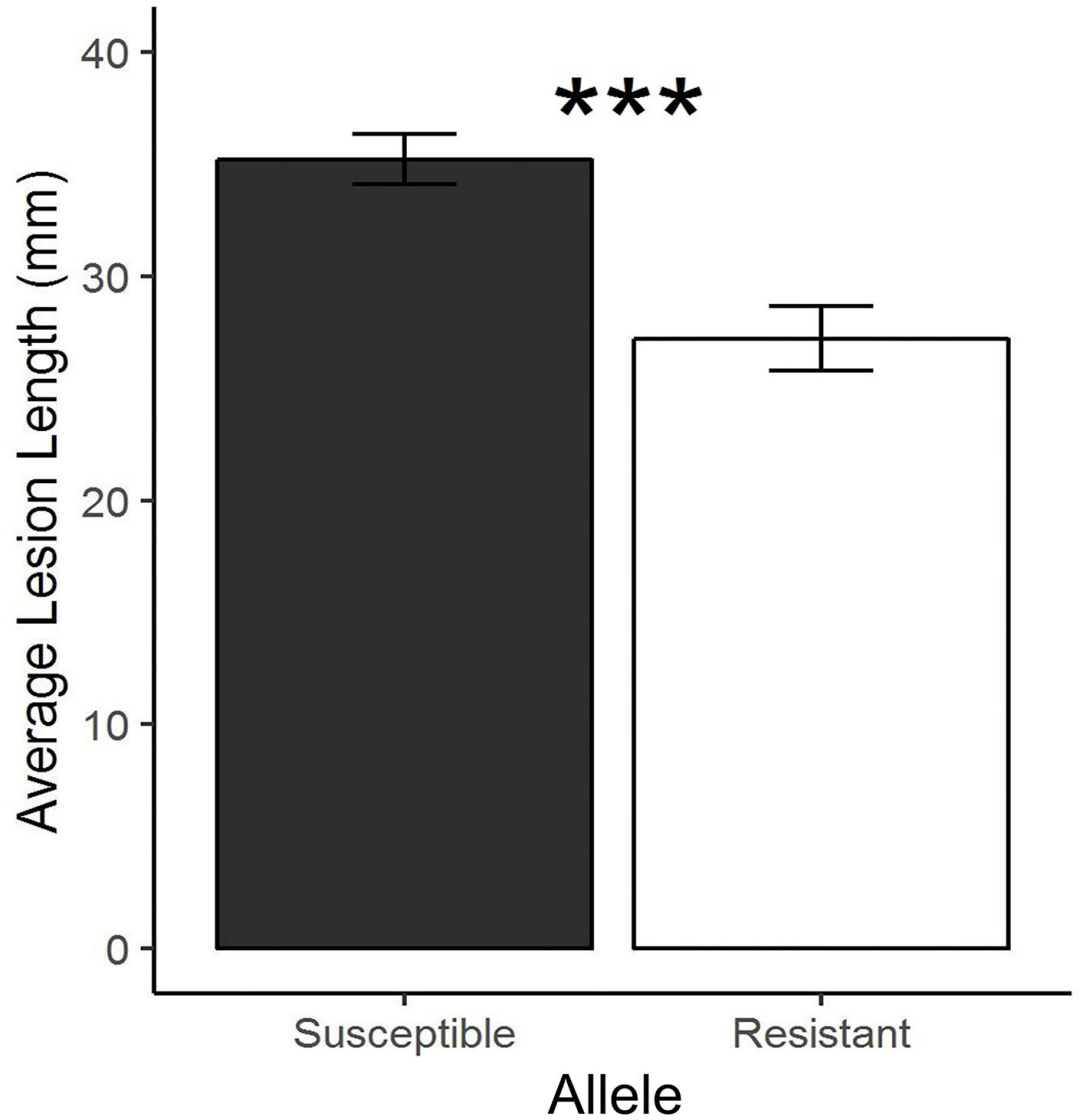
Average lesion length (±SE) between susceptible and resistant near-isogenic lines derived from a cross between OX20-8 and PI 427105B (***p < 0.001, Welch's *t*-test).

RNA-seq was performed on five resistant NILs, five susceptible NILs, and parental lines (PI 427105B and OX20-8) with inoculated (24 hai) and mock inoculated treatments. Successful inoculation was confirmed by lesion length at 7 dai (Figure 3). In total, the 204 samples resulted in 6.3 billion single-end reads. DEGs between alleles within treatment (resistant vs. susceptible alleles for both inoculated and mock treatments) and between treatment within allele (inoculated vs. mock for both resistant and susceptible alleles) were identified for each time point (Table 2; Supplementary Table S4). Across all comparisons, 4,749 unique genes were differentially expressed in the NILs based on the Williams 82 soybean reference genome (Wm82.a2.v1; Schmutz et al., 2010).

**Table 2:** Differentially expressed genes (DEGs) at 5% false discovery rate with log fold-change threshold of 2 between inoculated and mock inoculated resistance and susceptible near-isogenic lines derived from crosses between OX20-8 and PI 427105B and parental lines OX20-8 and PI 427105B, based on Williams 82 reference genome (Wm82.a2.v1; Schmutz et al., 2010).

		**3 hai**		**24 hai**		**48 hai**		**Total Unique**
	**Treatment** ^**†**^	**Up**	**Down**	**Up**	**Down**	**Up**	**Down**	**DEGs**
Williams 82 reference genome	Res. Mock vs. Sus. Mock	0	2	210	1	18	0	219
	Res. Inoc vs. Sus. Inoc	41	53	6	2	3	43	124
	Res. Inoc vs. Res. Mock	66	0	1,564	529	2,165	1,380	3,628
	Sus. Inoc vs. Sus. Mock	150	8	1,805	325	2,519	1,452	4,133
	PI 105B Mock vs. OX20-8 Mock	113	174	113	148	115	159	378
	PI 105B Inoc vs. OX20-8 Inoc	104	169	120	123	182	273	546
	PI 105B Inoc vs. PI 105B Mock	2	0	736	5	968	26	1,128
	OX20-8 Inoc vs. OX20-8 Mock	86	0	1,090	20	2028	586	2,771
*De novo* transcriptome	Res. Mock vs. Sus. Mock	1	2	25	9	45	31	110
	Res. Inoc vs. Sus. Inoc	100	75	11	10	132	1,525	275
	Res. Inoc vs. Res. Mock	398	28	7,172	1,136	1,1081	2,740	16,182
	Sus. Inoc vs. Sus. Mock	307	11	8,517	1,092	19,385	3,942	25,125

† *Inoculation with P. sojae isolate 1.S.1.1*.

Genes differentially expressed within a single NIL allele (resistant or susceptible) following inoculation may represent the plant's general response to pathogen attack rather than a specific resistance or susceptibility response. Overall, inoculation influenced gene expression more than NIL allele (Table 2). For the resistant NILs, 66, 2,093, and 3,545 DEGs were identified in response to inoculation at 3, 24, and 48 hai, respectively (Supplementary Figure S3). Similarly, for the susceptible NILs, 158, 2,130, and 3,971 DEGs were identified in response to inoculation at 3, 24, and 48 hai, respectively (Supplementary Figure S4). There were significant overlaps among these inoculation-response DEGs in the resistant and susceptible NILs, with 23, 80, and 2,934 inoculation response DEGs shared at 3, 24, and 48 hai, respectively. We looked at enrichment with AgriGO, DAVID, and KEGG pathways, with similar findings for each. Genes differentially expressed in response to inoculation within both the resistant and susceptible NIL introgressions, respectively, were enriched for Gene Ontologies associated with general defense response, such as oxidation reduction, protein phosphorylation, protein serine–threonine kinase activity, and response to stimulus (AgriGO, Supplementary Table S5; DAVID, Supplementary Table S6). Similarly, plant–pathogen interaction, MAPK signaling, and phenylpropanoid biosynthesis KEGG pathways were upregulated for both resistant and susceptible NILs (Supplementary Table S7).

**Figure 4:**
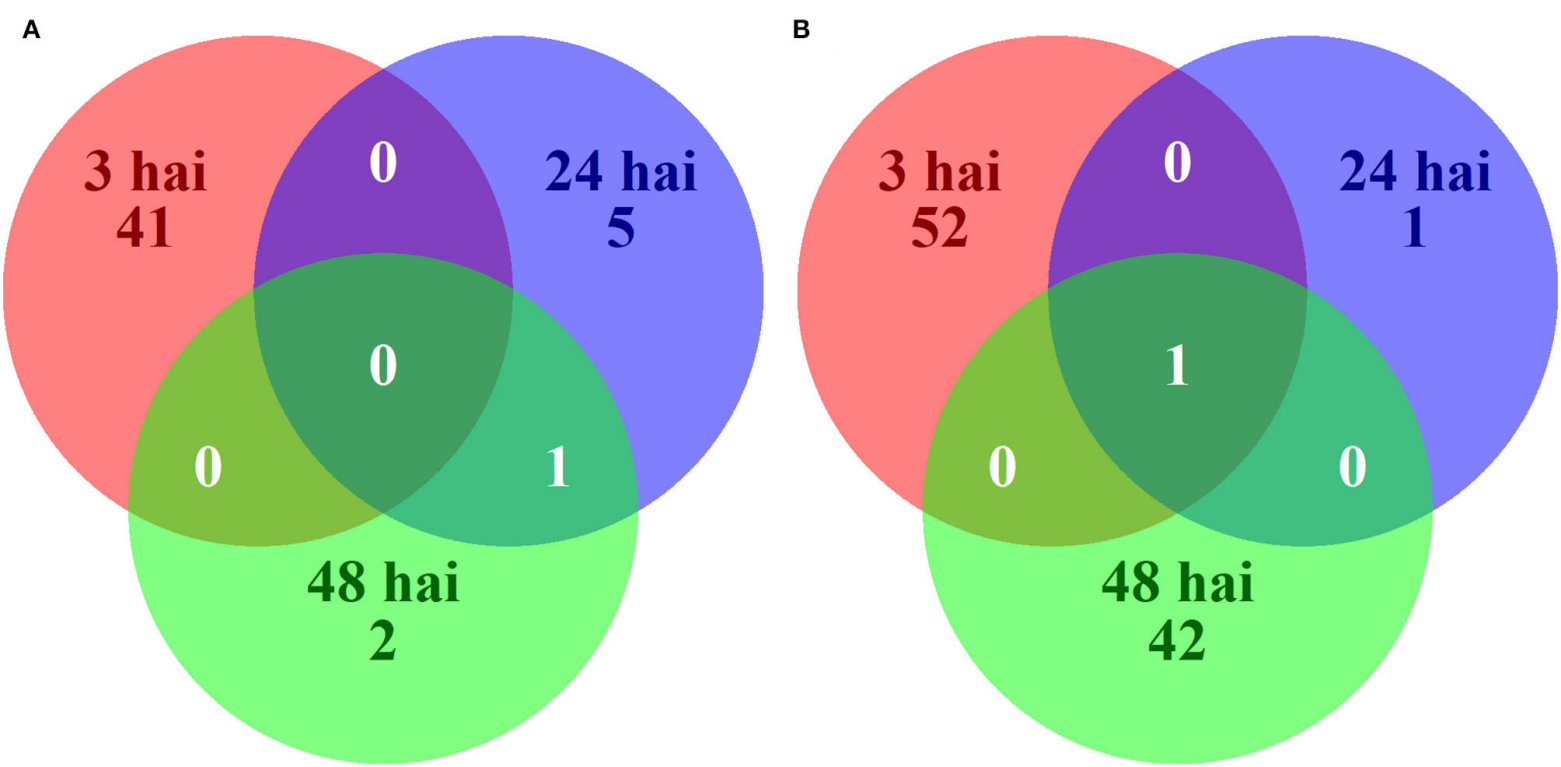
Venn diagram of 145 unique genes significantly upregulated **(A)** or downregulated **(B)** in resistant (PI 427105B allele) as compared to susceptible (OX20-8 allele) near-isogenic lines at 3, 24, and 48 h after inoculation (hai) with *P. sojae* isolate 1.S.1.1.

Differences in gene expression between resistant and susceptible NILs after inoculation with *P. sojae* may be the signals of induced resistance or susceptibility. Across all time points, there were 145 unique DEGs between resistant and susceptible NILs following inoculation with *P. sojae*. Among the 145 DEGs, 49 were expressed at higher levels in resistant NILs, and 96 were expressed at higher levels in susceptible NILs (Figure 4). KEGG pathway enrichment showed a significant downregulation of pathways in the resistant NILs compared to the susceptible NILs following inoculation. Among the pathways significantly downregulated in resistant lines are the glutathione (GSH) metabolism pathway at 3 hai and oxidative phosphorylation pathway at 24 hai (Table 3). DAVID highlighted the enrichment of upregulated genes in the cutin, suberin, and wax biosynthesis pathways at 3 hai (Supplementary Table S6).

**Table 3:** KEGG pathways significantly downregulated at each time point in resistant (Res)near-isogenic lines (NILs) compared to susceptible (Sus) NILs under inoculated (Inoc; *P. sojae* isolate 1.S.1.1.) and mock inoculated (Mock) conditions.

**Comparison**	**Time point**	**Downregulated^**†**^ KEGG Pathway**	***p*-value**
Inoc Res vs. Inoc Sus	3	gmx00480 Glutathione metabolism	<0.0001
Inoc Res vs. Inoc Sus	3	gmx00196 Photosynthesis - antenna proteins	0.001
Inoc Res vs. Inoc Sus	24	gmx01200 Carbon metabolism	<0.0001
Inoc Res vs. Inoc Sus	24	gmx00040 Pentose and glucuronate interconversions	0.0003
Inoc Res vs. Inoc Sus	24	gmx01230 Biosynthesis of amino acids	0.0012
Inoc Res vs. Inoc Sus	48	gmx04136 Autophagy - other	<0.0001
Inoc Res vs. Inoc Sus	48	gmx04144 Endocytosis	0.0004
Inoc Res vs. Inoc Sus	48	gmx00280 Valine, leucine and isoleucine degradation	0.0004
Inoc Res vs. Inoc Sus	48	gmx04016 MAPK signaling pathway - plant	0.0009
Inoc Res vs. Inoc Sus	48	gmx00010 Glycolysis / Gluconeogenesis	0.0042
Inoc Res vs. Inoc Sus	48	gmx00071 Fatty acid degradation	0.0043
Inoc Res vs. Inoc Sus	48	gmx04130 SNARE interactions in vesicular transport	0.0043
Inoc Res vs. Inoc Sus	48	gmx00190 Oxidative phosphorylation	0.005
Inoc Res vs. Inoc Sus	48	gmx03060 Protein export	0.0079
Inoc Res vs. Inoc Sus	48	gmx03050 Proteasome	0.0079
Inoc Res vs. Inoc Sus	48	gmx01200 Carbon metabolism	0.008
Mock Res vs. Mock Sus	24	gmx00196 Photosynthesis - antenna proteins	0.0002
Mock Res vs. Mock Sus	24	gmx00195 Photosynthesis	0.0002
Mock Res vs. Mock Sus	24	gmx03013 RNA transport	0.0019
Mock Res vs. Mock Sus	48	gmx00280 Valine, leucine and isoleucine degradation	0.0002

† *No KEGG pathways were upregulated in these comparisons*.

To explore potentially constitutive differences between resistant and susceptible NILs, differential gene expression analysis was performed between alleles within the mock treatment. In the absence of *P. sojae*, 225 unique DEGs were identified between NILs, with 222 expressed at higher levels in the resistant NILs and 3 expressed at higher levels in the susceptible NILs (Table 2). KEGG pathway analysis of all genes differentially expressed between resistant and susceptible mock highlighted pathways downregulated in resistant NILs, none obviously associated with resistance, but with roles in photosynthesis, RNA transport, and amino acid degradation (Supplementary Table S7). Yet, there was enrichment among DEGs for defense and stress response-related genes (AgriGO; Supplementary Table S5) as well as plant–pathogen interaction, response to stress, and plant hormone signal transduction pathways (DAVID; Supplementary Table S6). The specificity of the enrichment identified by DAVID to the 24 hai points toward a differential response to the mock inoculation rather than a constitutive difference between the resistant and susceptible NILs.

RNA-seq was also performed on parents OX20-8 (susceptible) and PI 427105B (resistant). Across all comparisons, 7,460 unique genes were differentially expressed. In response to *P. sojae* inoculation, 1,706 total genes were upregulated, and 31 were downregulated in PI 427105B, whereas 3,204 genes were upregulated and 606 were downregulated in OX20-8 (Table 2). Following inoculation, 546 unique genes were differentially expressed between lines (OX20-8 and PI 427105B) (Supplementary Figure S5). In the absence of *P. sojae*, 378 unique genes were differentially expressed between lines. In response to *P. sojae* inoculation, 1,706 total genes were upregulated, and 31 were downregulated in PI 427105B, whereas 3,204 genes were upregulated and 606 were downregulated in OX20-8 (Table 2). Thus, like the NILs, gene regulation was influenced more by inoculation than genotypic differences.

In total, seven DEGs colocalized with the narrowed 731-kb *QDRL-18* interval (Table 4). A total of six DEGs identified in comparisons between mock and inoculated treatments colocalized with the *QDRL-18* interval: within the resistant NILs, a gene encoding a putative oxidoreductase (*Glyma.18g026500*); within the susceptible NILs, genes putatively encoding a receptor-like protein kinase (*Glyma.18g026700*), chlorophyll a/b binding protein (*Glyma.18g028400*), and legume lectin domain (*Glyma.18g031400*); within both introgressions, genes putatively encoding a pollen protein (*Glyma.18g025200*) and member of the transferase family (*Glyma.18g029900*). One of the 145 unique DEGs identified between resistant and susceptible NILs following inoculation with *P. sojae* colocalized to the *QDRL-18* interval, and this was a gene putatively encoding a receptor-like protein kinase (*Glyma.18g026900*) expressed at higher levels in susceptible NILs at all three time points following inoculation. None of the 225 unique DEGs identified between resistant and susceptible NILs in the mock treatment colocalized with the *QDRL-18* interval. A number of two DEGs that were upregulated in OX20-8 following inoculation colocalized with *QDRL-18*: *Glyma.18g026900*, the gene putatively encoding a receptor-like protein kinase and expressed at higher levels in susceptible NILs following inoculation with *P. sojae*, and *Glyma.18g026700*, another gene putatively encoding a receptor-like protein kinase. The reduced expression of *Glyma.18g026900* in inoculated resistant NILs as compared to inoculated susceptible NILs was confirmed *via* RT-qPCR at 3 hai, but no significant differences in expression were confirmed at the later time points *via* RT-qPCR (Supplementary Figure S6).

**Table 4:** Seven differentially expressed genes at 5% false discovery rate with log-fold change threshold Quantitative disease resistance loci (QDRL) analysis Quantitative disease resistance loci (QDRL) analysis of 2 in near-isogenic lines based on Williams 82 reference genome (Wm82.a2.v1; Schmutz et al., 2010) that colocalize with the narrowed 731-kb QDRL-18 interval.

					**Treatment** ^**†**^					
**Gene ID** ^**‡**^	**PFAM Annotation**	**Res. Inoc vs. Sus. Inoc**			**Res. Inoc vs. Res. Mock**			**Sus. Inoc vs. Sus. Mock**		
		**3**	**24**	**48**	**3**	**24**	**48**	**3**	**24**	**48**
*Glyma.18g025200*	Pollen proteins Ole e I like					↓	↓			↓
*Glyma.18g026500*	2OG-Fe(II) oxygenase superfamily						↑			
*Glyma.18g026700*	Protein kinase domain									↑
*Glyma.18g026900*	Protein kinase domain	↓	↓	↓						
*Glyma.18g028400*	Chlorophyll A-B binding protein									↓
*Glyma.18g029900*	Transferase family					↑	↑			↑
*Glyma.18g031400*	Legume lectin domain									↓

† *Inoculation with P. sojae isolate 1.S.1.1.; no annotated genes within the 731-kb interval were differentially expressed for the resistant mock vs. susceptible mock comparison*.

‡ *Wm82.a2.v1 https://www.soybase.org*.

*Arrows indicate changes in expression levels for a given contrast and time point*.

### Differential Expression of Novel Transcripts

Disease resistance loci often vary in gene copy number (McHale et al., 2012), and several studies have established that the genes conferring resistance may be absent from Wm82.a2.v1 (Meyer et al., 2009; Cook et al., 2012). Thus, a *de novo* transcriptome was assembled from all NIL samples (*n* = 180) to identify novel transcripts absent from Wm82.a2.v1. For each sample, three million reads were randomly selected prior to assembly, for a total of 540 million reads, to maximize the number of soybean (Wm82.a2.v1) transcripts represented by nearly full-length Trinity transcripts (Supplementary Figure S1) while reducing the computational load. This resulted in 324,277 transcripts representing 190,916 Trinity “genes,” with each true gene potentially being represented by multiple Trinity “genes.” Median transcript length was 602 bases, with an average length of 1,045 bases and a 90% overall alignment rate.

Differential gene expression analysis was performed as described previously, and 31,876 unique Trinity “genes” were differentially expressed across all comparisons (Table 2). The Trinity “genes” differentially expressed between resistant and susceptible NILs within the inoculated treatment were enriched for Gene Ontology terms associated with the following biological processes: cell wall organization, circadian rhythm, transposition, and oxidation-reduction. Trinity “genes” that were differentially expressed between introgressions in the mock treatment, representing constitutive differences, were involved in cell communication, cytoskeleton organization, nucleosome assembly, cell maturation, and ion transport (Supplementary Table S8). Of the 31,876 differentially expressed Trinity “genes” identified, 8,810 were novel to the *de novo* transcriptome as determined by BLASTN results. In total, there were 267 and 62 novel Trinity “genes” differentially expressed between NIL introgressions within the inoculated and mock treatments, respectively (Supplementary Table S9). Whereas these represent potential candidate genes for *QDRL-18*, in this study, they have not been anchored to the *QDRl-18* interval.

### Sequence Analysis of *Glyma.18g026900*

As the only gene differentially expressed between resistant and susceptible NILs at any time point and localized to the reduced *QDRL-18* interval, we identified *Glyma.18g026900* as the most likely candidate gene for *QDRL-18*. Sequence comparison of *Glyma.18g026900* (1,413-bp genic region as annotated in Wm82.a2.v1 and 355-bp upstream) DNA derived from susceptible alleles (including Williams 82, OX20-8 and a susceptible NIL) and resistant alleles (including PI427106, PI 427105B, and a resistant NIL derived from PI427105B) revealed 89 SNPs and 4 deletions and one frameshift (Supplementary FASTA File). All sequences from resistant individuals were identical to each other as were all sequences from susceptible individuals. We analyzed this sequence for evidence of possible functional variation. *Glyma.18g026900* is predicted to encode a 470 amino acid serine–threonine protein kinase (STK) with its highest similarity in the *Arabidopsis thaliana* genome (Araport11; Cheng et al., 2017) to CRINKLY 4-related protein 3 (AtCCR3) (56% identity; BLASTP). *Glyma.18g026900* is a member of an 11 gene subfamily most closely related to AtCCR3 in soybean (Supplementary Figure S7).

**Figure 5:**
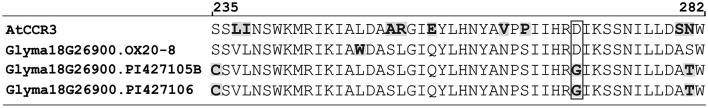
Amino acid alignment of the predicted activation site including *Glyma.18G026900* from soybean cv. OX20-8 (susceptible) and PI 427105B (resistant), and the CRINKLY 4-related protein 3 from *Arabidopsis thaliana* (AtCCR3-Q9LY50). Shaded areas highlight the differences among sequences whereas the rectangle denotes the predicted proton acceptor for the putative serine–threonine protein kinase activation site.

Within the coding region, a total of 74 SNPs, 4 indels, and a C-terminal frameshift were noted whereas 65 SNP represented non-synonymous changes (Supplementary Figure S8). In pairwise comparisons of the two alleles, the N-terminal and C-terminal domains of the protein had a higher rate of non-synonymous substitutions (dN) compared to the rate of synonymous substitutions (dS), with the significant evidence of positive selection in the C-terminal domain (Table 5). However, only eight of non-synonymous SNPs are in conserved regions, defined as ≥75% conservation among related proteins (Phyre2; Kelley et al., 2015). Of these eight non-synonymous polymorphisms, D270G is predicted to be within one of the protein's five regions with sufficient flexibility (PROFbval 31-70) to serve as an enzymatic activation site (Figure 5) (Bernhofer et al., 2021) and aligns to the proton acceptor within the activation site functioning of AtCCR3 (The UniProt Consortium, 2021). Susceptible-derived (from Williams 82, OX20-8, or susceptible NILs) versions of *Glyma.18g026900* share the aspartic acid (D) residue predicted to function as a proton acceptor in AtCCRs; however, resistant-derived versions of *Glyma.18g026900* code for a glycine (G) at this position (Figure 5). In the resistant-derived sequence, no activation site was predicted by the InterPro Scan5 (Jones et al., 2014).

**Table 5:** Comparison of synonymous substitution rate (dS) to nonsynonymous substitution rate (dN) between translated sequences of *Glyma.18G026900* from PI 427105B vs. OX20-8.

**Protein Region^**†**^**	**bp**	**dN- dS**	***p*-value^**‡**^**
N-terminus	1–434	0.63	0.26
PK domain	435–1,272	−1.37	1.00
PK activation site	795–834	−1.02	1.00
C-terminus	1,273–1,454	2.08	0.02

† *InterProScan5 (Jones et al., 2014)*.

‡ *Z-test of selection performed using MEGA11 (Tamura et al., 2021). P < 0.05 is indicative that the alternative hypothesis of positive selection (dN > dS) is supported over the hypothesis of neutrality (dN = dS)*.

A total of two of the four indels are 3 nt in length and code for single amino acid indels. Indel L468_I469 del is 5 nt and is predicted to result in a frameshift at the C-terminus with the resistant-derived allele coding for 12 additional amino acids and the susceptible-derived (from Williams 82, OX20-8, or susceptible NILs) allele terminating one amino acid after the indel. The largest indel coded for 29 amino acids (A84_A114del) within the backbone of the predicted STK, with the susceptible-derived sequences predicted to encode an additional 29 amino acids relative to the PI 427105B-derived sequences. The 29 amino acid fragment has a high concentration of serine (8) and threonine (1) residues and is predicted to be exposed for DNA/RNA binding. The absence of this region in resistant-derived sequences represents a loss of 17% of serine and 5% of threonine residues as compared to the susceptible. Predicted promoter regions were relatively conserved with a single C/G variant between resistant-derived sequences (C) and susceptible-derived sequences (G). Thus, whereas significant portions of the protein exhibit the evidence of positive selection, we predicted that the D270G and/or the A84_A114del polymorphisms are the sequence variations most likely to cause functional change.

### JA and SA Accumulation 24 h After Inoculation

**Figure 6:**
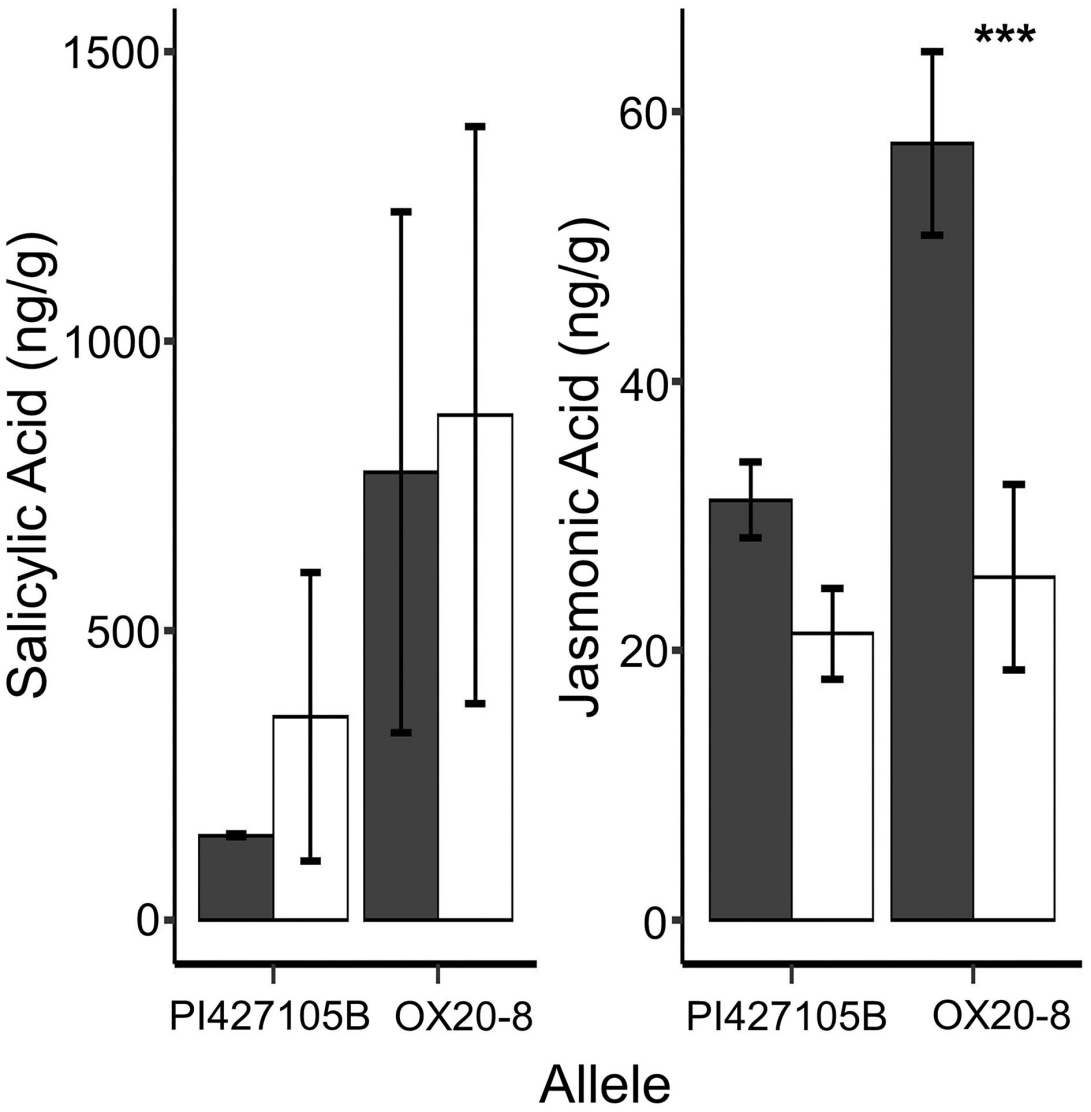
Average concentration (ng/g) for salicylic (SA) and Jasmonic (JA) acid root crown (24 hai). Black (Inoc-zoospores) and white (Mock-water) illustrate treatments. Error bars represent the standard error (±SE) whereas asterisk above the bars denote significance level (***p < 0.001, Fisher's protected *t*-test).

The Arabidopsis homolog of *Glyma.18g026900*, AtCCR3, is involved in regulation of JA, and changes in phytohormone levels are among the earliest modes of action correlated with plant defense (Pieterse et al., 2009). JA is often described as a defense signal against necrotrophic pathogens and herbivores, whereas salicylic acid (SA) is usually associated with contributing resistance to biotrophic and hemibiotrophic pathogens (Cui et al., 2010; Pan et al., 2016). Therefore, we used UPLC-QqQ-MS to quantify the SA and JA in the target zone of inoculation or “root crown” from inoculated and mock inoculated seedlings in a tray test 24 hai. SA was significantly affected by allele and treatment, but not their interaction (Supplementary Table S10). Mock treatments and susceptible NILs had lower SA accumulation in the root crown at 24 hai (Figure 6). Interestingly, JA concentration had a significant allele by treatment interaction with a specific and significant increase in JA accumulation in the root crown of inoculated susceptible NILs (24 hai; Figure 6).

## Discussion

### *QDRL-18* Was Narrowed to a 3.1 cm Genetic Interval and Has Potential for Agronomic Impact

Identifying the gene(s) underlying resistance loci is an important step in the ongoing effort to dissect the molecular mechanisms and biological bases of quantitative disease resistance. In this work, we were able to reduce the *QDRL-18* physical interval by 40%. Providing sufficient recombination occurs, this is expected to decrease the likelihood of introducing genetically linked deleterious alleles during marker-assisted selection (MAS) of the QDRL (Thomas et al., 1998; Kopisch-Obuch et al., 2005). However, with 82 genes remaining in this interval, pinpointing a specific candidate gene remains a challenging task (St.Clair, 2010). Near-isogenic material is a valuable tool for identifying transcriptional and biochemical differences associated with a specific genetic region of interest (Kim et al., 2011; Peiffer et al., 2012; Häffner et al., 2014; Lee et al., 2017; Wang et al., 2017). Whereas previous studies have identified potential mechanisms of partial resistance to *P. sojae* by contrasting resistant and susceptible germplasm (Wang et al., 2010), the comparison of NILs allows for the identification of functional pathways as well as candidate genes for a specific resistance locus. Therefore, we combined our genetic information with RNA-seq analysis of NILs derived from an OX20-8 × PI 427105B RIL population to identify putative pathways involved in resistance and candidate genes specifically associated with *QDRL-18*.

The integration of mapping and gene expression studies has emerged as a potential method for candidate gene identification. However, this approach requires that the gene(s) underlying resistance are differentially expressed between resistant and susceptible plants. For instance, it would fail to detect the wheat (*Triticum aestivum* L.) gene *Lr34*, which putatively encodes an adenosine triphosphate-binding cassette (ABC) transporter that confers resistance to multiple leaf pathogens in wheat (Krattinger et al., 2009). In the case of *Lr34*, resistant and susceptible NILs do not exhibit gene expression differences, and no sequence variation exists within 2 kb of the putative promoter region (Krattinger et al., 2009). Despite this restriction, differential gene expression analysis has been successfully used to identify the candidate genes for iron efficiency (Peiffer et al., 2012; Atwood et al., 2014), bacterial leaf pustule resistance (Kim et al., 2011), resistance to soybean aphid (Lee et al., 2017), and resistance against cyst nematodes in soybean (Jiang et al., 2021). Thus, combined transcriptomic and linkage analyses represent a viable tool for identifying potential gene(s) underpinning *QDRL-18*.

In the literature, no yield reductions in soybean cultivars with partial resistance or single *Rps* alleles have been shown when exposed to low or no disease pressure (Dorrance et al., 2003). Our data are in line with these previous findings, with no significant effect of allele in environments with less to no disease pressure from *P. sojae*. NIL sets with the resistant introgression consistently outperformed or matched susceptible NILs in conditions with reduced disease pressure. Moreover, the resistant allele of *QDRL-18* increased yield by an average of 21% in fields with history of PRR disease. These findings indicate that the resistance allele of *QDRL-18* may be an excellent breeding target.

### Gene Ontology Enrichment of DEGs Hints at Roles of GSH Metabolism Underpinning *QDRL-18-*Mediated Resistance

Consistent with previous studies, vast transcriptional reprogramming occurred as a result of inoculation with *P. sojae* (Zhou et al., 2009; Wang et al., 2010). Greater than 10-fold, more genes were differentially expressed as a result of inoculation as compared to the *QDRL-18* allele. Yet, those genes that were differentially expressed between resistant and susceptible can elucidate the pathways involved in QDR (Chandra et al., 2016; Li et al., 2021). In comparison with resistant NILs, susceptible NILs in this study exhibited the upregulation of genes within the GSH metabolism pathway at 3 hai. The upregulation of genes within the GSH metabolism pathway is also found at 3 and 24 hai in response to inoculation in the susceptible NILs, but is not found until 48 hai in response to inoculation in the resistant NILs.

Glutathione is a major plant antioxidant (Noriega et al., 2012; Aslam et al., 2021), and accumulation and redox status of GSH is associated with a plant's ability to tolerate stress through the GSH reduction of H_2_O_2_ and reactive oxygen species when a plant is experiencing oxidative stress (Rausch et al., 2007). Additionally, Chen et al. (2017) proposed a model for crosstalk through GSH-mediated redox and defense-related signaling pathways. While the exact contribution of GSH in JA signaling is unclear, upregulation of the JA pathway triggered by intracellular oxidation requires GSH accumulation (Han et al., 2013; Aslam et al., 2021). Plant defense hormones, including SA and JA, have been shown to regulate gene expression through H_2_O_2_ (Mur et al., 2006), and exogenous application of SA to soybean cell suspensions increases GSH, providing a potential substrate for the indirect crosstalk with GSH. Rapid accumulation of GSH in susceptible NILs may be a cause of or response to susceptibility to *P. sojae*. The resulting accumulation of GSH in susceptible NILs could prevent the production of H_2_O_2_ and impact plant defense hormone signaling.

### Variation Within an STK May Lead to QDR Through Perturbation of GSH and JA Pathways

There are many defense-related genes differentially expressed between the resistant and the susceptible NILs in both the mock inoculated and the inoculated, yet only the 82 genes located within the narrowed *QDRL-18* interval represent positional candidates for controlling this source of resistance. A total of 329 “Trinity genes” were also differentially expressed between the resistant and the susceptible NILs and absent from the Williams 82 reference genome. These may represent further candidate genes; however, in the absence of physical positions relative to the *QDRL-18* interval, it is not feasible to further consider these genes as the positional candidates for *QDRL-18*. Of the 82 positional candidate genes, only *Glyma.18g026900*, putatively encoding a receptor-like kinase, specifically a STK with sequence similarity to *AtCCR3*, was differentially expressed between resistant and susceptible NILs, with a higher level of expression in the susceptible NILs confirmed *via* both RNA-seq and RT-qPCR at 3 hai. While RNA-seq also showed higher expression in the inoculated susceptible NILs at 24 and 48 hai, these later time points were not confirmed by RT-qPCR. The lack of confirmation between the two methods may be due to noise in the RT-qPCR data or to the significant sequence variation between the reference genome and PI 427105B allele, which could affect mapping to the genome and RNA-seq based expression counts. In either case, *Glyma.18g026900* represents a positional candidate with extensive polymorphism between resistant and susceptible NILs and, perhaps limited, differential expression.

In *Glyma.18g026900*, the increased level of expression in susceptible compared to resistant genotypes suggests that the putative STK could possibly be acting as a susceptibility factor. In eukaryotes, kinases participate in a wide range of biological reactions such as regulators of plant growth and development, but often are associated with plant–pathogen interactions (Xing et al., 2002). Such interactions are variable, with receptor-like protein kinases sometimes acting as pattern recognition receptors that detect microbe-associated molecular patterns (MAMPs) as well as damage-associated molecular patterns (DAMPs) to initiate an immune response and actively forms part of the stress signaling transduction through phosphorylation (Lindner et al., 2012; Zhang et al., 2013; Zipfel, 2014; Máthé et al., 2019). Such enzymes often either autophosphorylate or catalyze the transfer of a phosphate group from ATP to a protein substrate residue, such as serine or threonine amino acids (Hardie, 1999). However, kinases functioning in this manner would be expressed at higher levels in resistant lines following inoculation, yet, for *Glyma.18g026900*, we observed the opposite.

Arabidopsis *Crinkly 4* (*AtCR4*) is a well-studied family member of *AtCCR3*, the Arabidopsis homolog of *Glyma.18g026900*. AtCR4 encodes an STK with reported roles in plant development, defense, regulation of JA synthesis and root morphology (Zereen and Ingram, 2012; Czyzewicz et al., 2016). Aligning with our prediction of reduced functionality of the PI 427105B allele of *Glyma.18g026900*, knockouts of *AtCR4* resulted in reduced susceptibility to the necrotrophic pathogen, *Botrytis cinerea* (Zereen and Ingram, 2012) as well as increased expression of genes critical for JA biosynthesis (Bell et al., 1995). Often SA and JA pathways have a well-supported antagonistic relation among plants, where SA has been shown to decrease or may stay constant in response to an increase in JA (Kunkel and Brooks, 2002; Wang et al., 2020). In the soybean *P. sojae* pathosystem, exogenous application of SA was shown to have a protective effect (Sugano et al., 2013). In incompatible reactions to *P. sojae*, the JA pathway was suppressed (Lin et al., 2014). Indeed, SA, rather than JA, is generally the phytohormone associated with contributing resistance to hemibiotrophic pathogens (Robert-Seilaniantz et al., 2007; Pan et al., 2016). However, a cultivar with high levels of quantitative resistance was not affected by high levels of auxin nor its precursors; JA was proposed as playing a role in the later stages of infection (Stasko et al., 2020).

In addition to differential expression between the two alleles, there are major differences in the coding sequences suggesting that *Glyma.18g026900* derived from PI 427105B may not possess the same function as *Glyma.18g026900* derived from susceptible lines. First, a D270G substitution is within the predicted activation site, with the arginine to glycine change possessing a high dissimilarity index (33%) with regard to polarity and net charge (Sneath, 1966). Second, the A84_A114del results in the loss of 8 serines and 1 threonine in lines with the PI 427105B allele. Loss of serine and threonine amino acids can decrease the peptide's ability to autophosphorylate (Klaus-Heisen et al., 2011; Taylor et al., 2013), and alteration of the activation site impacts kinase regulation, phosphorylation, and chemical activity (Klaus-Heisen et al., 2011; Wang and Cole, 2014). Reduced kinase activity of *Glyma.18g026900* derived from PI 427105B may have resulted in the downregulation of oxidative phosphorylation pathways observed in the resistant NILs. Finally, the excess of non-synonymous mutations in the C-terminal end of the protein is indicative of positive selection, characteristically found in proteins involved in plant–pathogen interactions. Overall, these mutations suggest that, compared to the OX20-8 derived allele of *Glyma.18g26900*, the PI 427105B-derived allele of *Glyma.18g026900* may have reduced functionality as a kinase or different physical interactions with other plant or pathogen proteins.

We found specific and significant increase in JA accumulation in the inoculated treatment of susceptible NILs at 24 hai. It is unclear whether the accumulation of JA is due to the successful colonization of *P. sojae* or if, like some of our earlier studies (Stasko et al., 2020), increased JA aids in successful colonization by *P. sojae*. While our data do not show a concomitant response in SA accumulation, the clear increase in JA in response to inoculation in susceptible NILs combined with the known antagonistic relation could implicate *QDRL-18* as a negative regulator of plant immunity. Similarly, SA concentrations in root tissues decreased during initial phases of *Phytophthora medicaginis* infection in susceptible *Cicer arietinum* (chickpea) whereas JA concentrations are induced (Coles et al., 2022). Limited studies are related to JA, GSH, and response to biotic stress; however, Sirhindi et al. (2015) revealed that in soybean experiencing abiotic stress, JA can inhibit peroxidase activity by enhancing the GSH antioxidant machinery, allowing us to draw a loose link between the putative reduced function of *Glyma.18g026900*, enrichment of GSH metabolism genes within DEGs, and increased JA accumulation data. Thus, we speculate that *Glyma.18g026900* functions to enhance susceptibility to *P. sojae* through the induction of JA and GSH; however, further transgenic complementation studies need to be conducted to prove this functionality.

## Conclusion

In this study, the *QDRL-18* locus was reduced to a 731-kb interval, containing 82 predicted genes. The resistant allele of *QDRL-18* was shown to have no evidence of yield drag in fields lacking disease pressure, but significantly increases yield under disease conditions. It is expected to be a useful source of resistance in cultivar development. Among the 82 genes, only seven were differentially expressed following *P. sojae* inoculation. *Glyma.18g026900* was differentially expressed between resistant and susceptible NILs, possesses potentially functional sequence variation between alleles derived from resistant and susceptible lines, and represents an excellent candidate gene. The functions of homologs of *Glyma.18g026900*, combined with the increased JA and downregulated GSH metabolism in inoculated susceptible NILs, point toward *QDRL-18* potentially acting as a susceptibility factor. The narrowed *QDRL-18* region will greatly facilitate marker-assisted selection to increase levels of partial resistance to *P. sojae* by providing more closely linked markers. Further functional analysis of differentially expressed candidate genes can contribute to our understanding of the genes conditioning quantitative resistance and potential roles.

## Data Availability Statement

The datasets presented in this study can be found in online repositories. The names of the repository/repositories and accession number(s) can be found below: Data analyzed in this study can be found in a public repository GitHub: https://github.com/vargas-garcia/Glyma.18G026900.git and NCBI-Sequence Read Archive (PRJNA811603).

## Author Contributions

SL and MM developed all plant materials used in this study. MG contributed to library preparation and analysis of RNA-seq experiment. SK performed field trials, genotyping, genetic map construction, mapping, pathogen inoculation, RNA isolation, and bioinformatics analyses. CV-G performed field trials, pathogen inoculation assays, DAVID enrichment analysis, Sanger sequence analysis, and salicylic and jasmonic acid analysis. LM conceived the experiments and supervised the analyses. The paper was written by SK, CV-G, LM, and AD. All authors have read and approved the final manuscript.

## Funding

Salaries and research support were provided by The Ohio State University, United Soybean Board (Projects 2120-172-0132, 2020-172-0138, 1920-172-0110, 1720-172-0125) the Ohio Soybean Council (20-R-10, 19-R-21), the National Institute of Food and Agriculture, U.S. Department of Agriculture (Project 5030-21000-066-00D), Hatch projects Development of Disease Management Strategies for Soybean Pathogens in Ohio OHO01303, and Genetic Analysis of Soybean Added-Value Traits and Soybean Variety Development for Ohio OHO01279. SK was supported by the American Society of Agronomy United Soybean Board Fellowship.

## Author Disclaimer

Mentioning the trade names or commercial products in this publication is solely for the purpose of providing specific information and does not imply recommendation or endorsement by USDA. USDA is an equal opportunity provider and employer.

## Conflict of Interest

The authors declare that the research was conducted in the absence of any commercial or financial relationships that could be construed as a potential conflict of interest. The reviewer WU declared a shared affiliation with the authors MM and MG to the handling editor at the time of review.

## Publisher's Note

All claims expressed in this article are solely those of the authors and do not necessarily represent those of their affiliated organizations, or those of the publisher, the editors and the reviewers. Any product that may be evaluated in this article, or claim that may be made by its manufacturer, is not guaranteed or endorsed by the publisher.
