# Supplementary material for: Identification of Candidate Genes for a Major Quantitative Disease Resistance Locus From Soybean PI 427105B for Resistance to Phytophthora sojae
Source: Front Plant Sci. 2022 Jun 14;13:893652. doi: 10.3389/fpls.2022.893652 (PMC9237613; doi:10.3389/fpls.2022.893652)
Supplement: Supplementary file 12 [file Image_1.PDF]

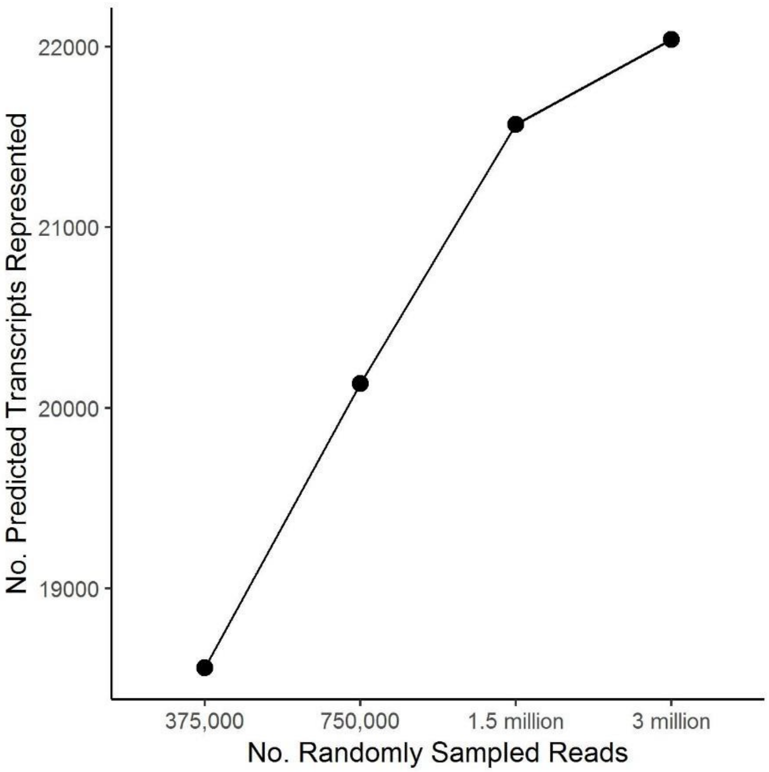

Supplementary Figure 1. Number of predicted soybean (Wm82.a2.v1) transcripts represented by nearly full-length Trinity transcripts having greater than 80% coverage for de novo transcriptome assemblies read randomly sampled reads at four levels for RNA-seq libraries from near isogenic lines (NILs) derived from OX20-8 × PI 427105B.
