# Supplementary material for: Identification of Candidate Genes for a Major Quantitative Disease Resistance Locus From Soybean PI 427105B for Resistance to Phytophthora sojae
Source: Front Plant Sci. 2022 Jun 14;13:893652. doi: 10.3389/fpls.2022.893652 (PMC9237613; doi:10.3389/fpls.2022.893652)
Supplement: Supplementary file 13 [file Image_2.PDF]

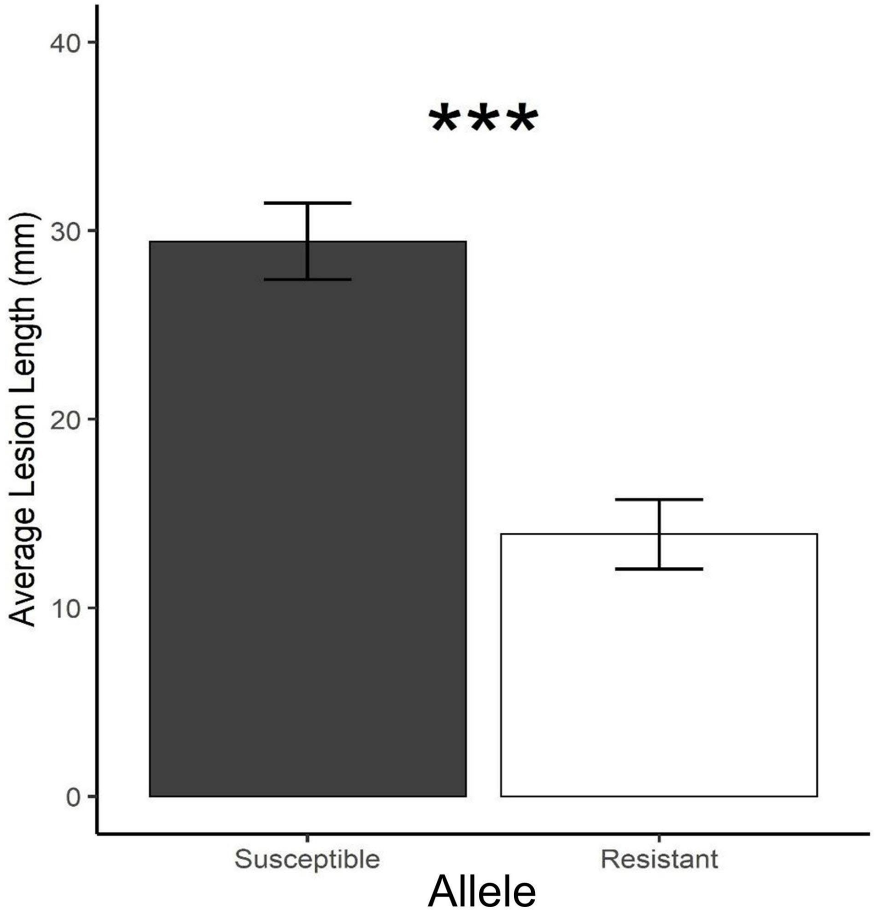

Supplementary Figure 2. Average lesion length for a <u>second biological repetition</u> for salicylic and jasmonic acid testing (±SE) between susceptible and resistant near-isogenic lines (NILs) derived from a cross between OX20-8 and PI 427105B (1.S.1.1) (***P < 0.001, Welch's t-test)
