# Supplementary material for: Identification of Candidate Genes for a Major Quantitative Disease Resistance Locus From Soybean PI 427105B for Resistance to Phytophthora sojae
Source: Front Plant Sci. 2022 Jun 14;13:893652. doi: 10.3389/fpls.2022.893652 (PMC9237613; doi:10.3389/fpls.2022.893652)
Supplement: Supplementary file 14 [file Image_3.PDF]

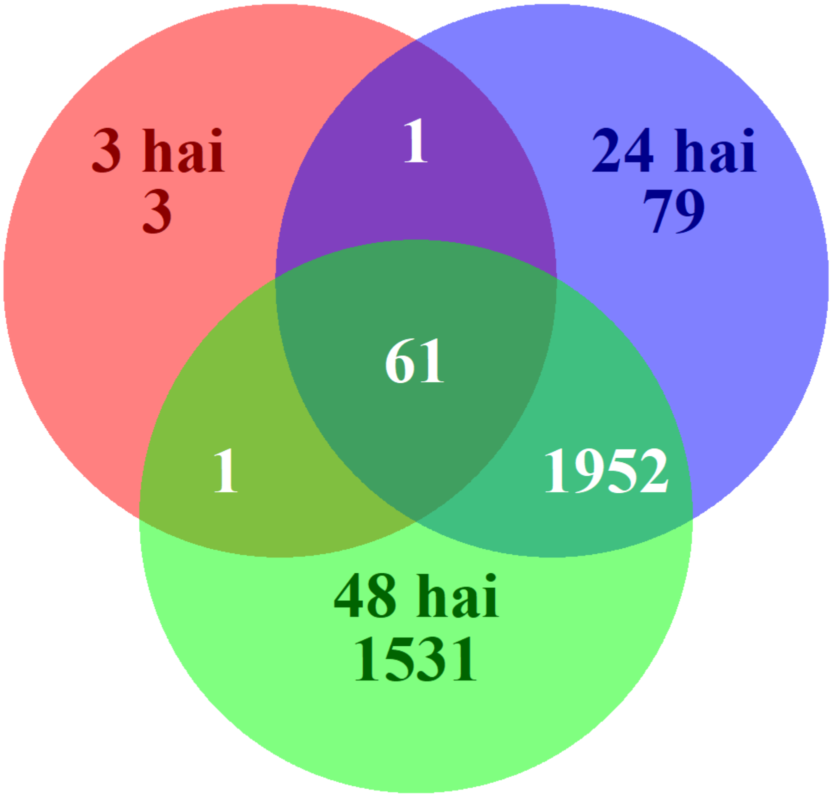

Supplementary Figure 3. Venn diagram of differentially expressed genes between inoculated and mock treatments for resistant near isogenic lines 3, 24, and 48 hours after inoculation (hai) with *Phytophthora sojae* (1.S.S.1).
