# Supplementary material for: Identification of Candidate Genes for a Major Quantitative Disease Resistance Locus From Soybean PI 427105B for Resistance to Phytophthora sojae
Source: Front Plant Sci. 2022 Jun 14;13:893652. doi: 10.3389/fpls.2022.893652 (PMC9237613; doi:10.3389/fpls.2022.893652)
Supplement: Supplementary file 16 [file Image_5.PDF]

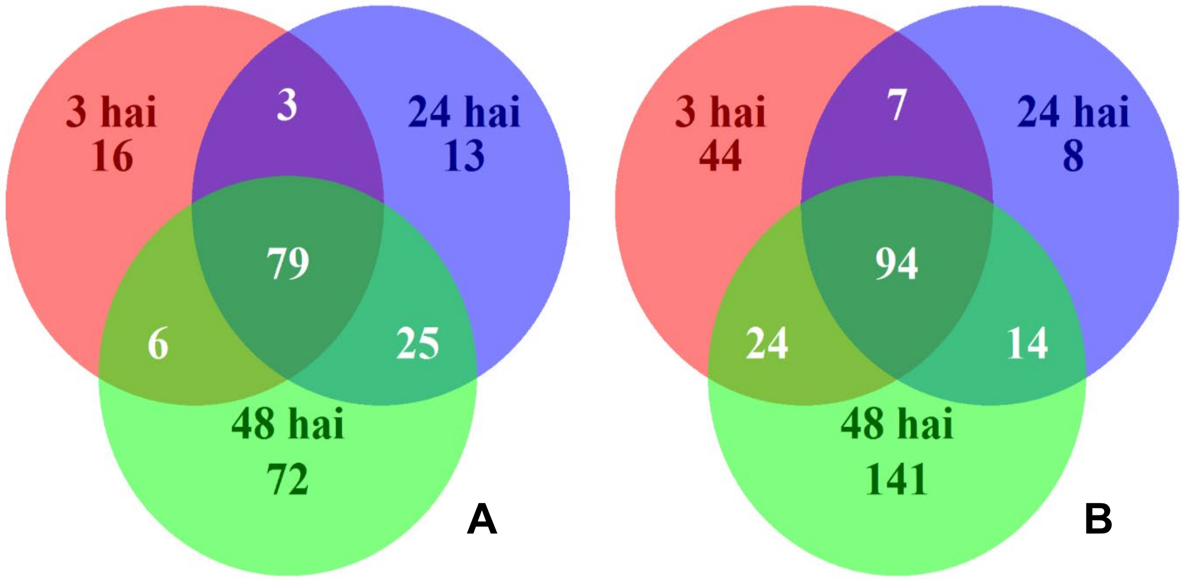

Supplementary Figure 5. Venn diagram of differentially expressed genes between OX20-8 (susceptible) and PI 427105B (resistant) following inoculation with *P. sojae* (1.S.1.1) upregulated **(A)** and downregulated **(B)** 3, 24, and 48 hours after inoculation (hai).
