# Supplementary material for: Identification of Candidate Genes for a Major Quantitative Disease Resistance Locus From Soybean PI 427105B for Resistance to Phytophthora sojae
Source: Front Plant Sci. 2022 Jun 14;13:893652. doi: 10.3389/fpls.2022.893652 (PMC9237613; doi:10.3389/fpls.2022.893652)
Supplement: Supplementary file 17 [file Image_6.PDF]

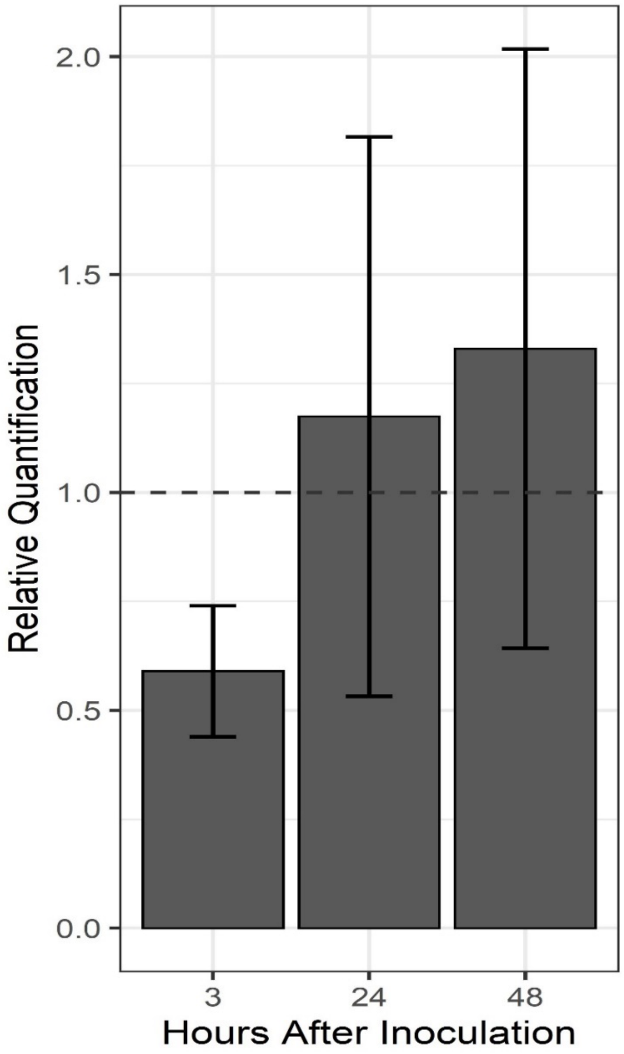

Supplementary Figure 6: Relative quantification ($2^{-\Delta\Delta Ct}$) of *Glyma.18G026900* transcripts at 3, 24, and 48 hours after inoculation (1.S.1.1) for inoculated resistant near isogenic lines (NILs) relative to inoculated susceptible NILs. Bars represent standard error of the mean.
