# Supplementary material for: Identification of Candidate Genes for a Major Quantitative Disease Resistance Locus From Soybean PI 427105B for Resistance to Phytophthora sojae
Source: Front Plant Sci. 2022 Jun 14;13:893652. doi: 10.3389/fpls.2022.893652 (PMC9237613; doi:10.3389/fpls.2022.893652)
Supplement: Supplementary file 18 [file Image_7.PDF]

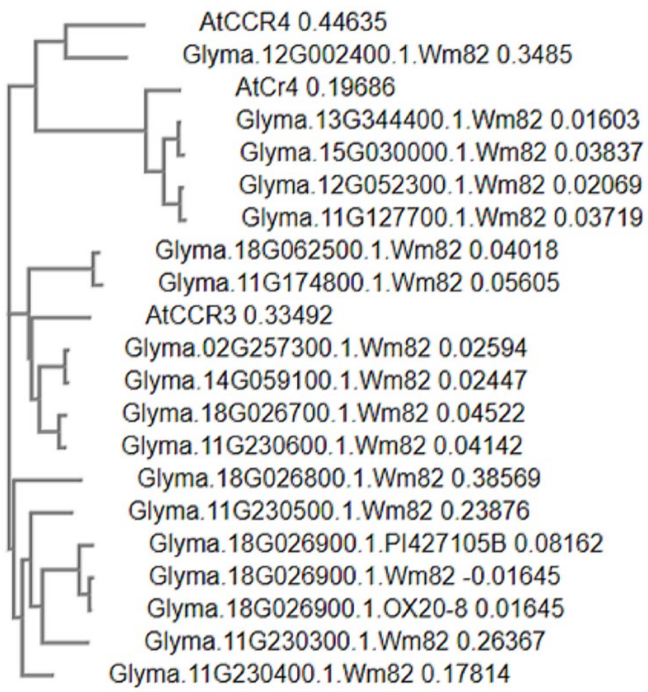

Supplementary Figure 7. Neighbor-joining phylogenetic tree from an amino acid alignment of Glyma.18G026900, the Arabidopsis homolog and paralogs AtCCR3, AtCCR4, and AtCR4, as well as paralogs from soybean (Wm82.a2.v1) built with Clustal Omega and EMBL Simple Phylogeny (Sievers and Higgins, 2014; Goujon et al., 2010).
