# Supplementary material for: Identification of Candidate Genes for a Major Quantitative Disease Resistance Locus From Soybean PI 427105B for Resistance to Phytophthora sojae
Source: Front Plant Sci. 2022 Jun 14;13:893652. doi: 10.3389/fpls.2022.893652 (PMC9237613; doi:10.3389/fpls.2022.893652)
Supplement: Supplementary file 19 [file Image_8.PDF]

```
            1                                                                                82
OX20-8      MDYEIAESTDKADITVDARSFSWAVDSAIKSETASILGESRIHSFCSVVDNAIRSRTASDLGTSPSPVHSLASAVANAMSSS
PI427105B   MDSEIVESTDMADITVDAKSFSWAVDSAIKSETASISGESRIHSFCSVVDNAIRSRTASDLGTSPSPVHSLASAVANAMSSS
Williams-82 MDYEIAESTDKADITVDARSFSWAVDSAIKSETASILGESRIHSFCSVVDNAIRSRTASDLGTSPSPVHSLASAVANAMSSS

..................................................................................

            83                                                              PK Domain   164
OX20-8      SAAPFSQLETSPSPVQSLASAVPSAMSRSKAAPFSQLEVVPNATRHVPARLFTWAELKAATNNFSPHNRIRHFGTFGFGYRG
PI427105B   SA-----------------------------APFSQLEVVPNATGLVPARLFTWSELKEATYNFSPHNRIRHFGSFEFVYRG
Williams-82 SAAPFSQLETSPSPVQSLASAVPSAMSRSKAAPFSQLEVVPNATRHVPARLFTWAELKAATNNFSPHNRIRHFGTFGFGYRG

..................................................................................

            165                                                                      246
OX20-8      KLVDGREVAIVKGTQFGKSEFAIFSRLDHRNLVGLVGYCENRDERLSVYEYMKNGLLRDCLHDKNNVDKDSSVLNSWKMRIK
PI427105B   KLVDGREVAIVKETPFGKSEFAIFSRLDHRNLVGLVGHCENRDERLSVYEYMKNGSLHDCLQDKNNVDKDCSVLNSWKMRIK
Williams-82 KLVDGREVAIVKGTQFGKSEFAIFSRLDHRNLVGLVGYCENRDERLSVYEYMKNGLLRDCLHDKNNVDKDSSVLNSWKMRIK

..................................................................................

                              Active Site
                                Domain
            247                                                                      328
OX20-8      IAWDASLGIQYLHNYANPSIIHRDIKSSNILLDASWTARVFDFELSFICPEPDDEYDEIIMGTMGYMCPEYMTRGVLTAKCD
PI427105B   IALDASLGIQYLHNYANPSIIHRGIKSSNILLDATWTARVFGFGWSFICPEPDGEYEERIVGTYGYISPEYAMWGVLTAKCD
Williams-82 IAWDASLGIQYLHNYANPSIIHRDIKSSNILLDASWTARVFDFELSFICPEPDDEYDEIIMGTMGYMCPEYMTRGVLTAKCD

..................................................................................

            329                                                                      410
OX20-8      VYGLGVVLLELLTGKRAILLESEEDGPPFYLVDFAVPPILAGDLVEILDSRVGPPDLKEAEALKILAHTAIRCVEEEPRQRP
PI427105B   VYALGVVLLELLTGKRVILPSE--DGEEPRLLDFAVPPILAGDLVEILDSRVGPPDVKEAEALKILAHTAIRCVEVEPRHRP
Williams-82 VYGLGVVLLELLTGKRAILLESEEDGPPFYLVDFAVPPILAGDLVEILDSRVGPPDLKEAEALKILAHTAIRCVEEEPRQRP

..................................................................................

            411                                                              482
OX20-8      TMNDIVVDLDRAFAICGSSLYDENFSYSIALPSHESNHMTPDPLDTDSCDIPIYEENTNS------------
PI427105B   TMNDIVVDLDRAFAICGSSLYDENFSYSNALPSHESNHMTPDPLDTDSCDIPIDEETLIIDFLNSLKHPSPR
Williams-82 TMNDIVVDLDRAFAICGSSLYDENFSYSIALPSHESNHMTPDPLDTDSCDIPIYEENTNS------------

..................................................................................
```

Supplementary Figure 8. Protein alignment for *Glyma.18G026900* gene depicting Susceptible (OX20-8), Resistant (PI427105B), and Williams-82 (Reference) translation. The "Black Lines" represents the serine/threonine-protein kinase predicted domains (*InterProScan 87.0*). The "Rectangle" denotes the predicted active catalytic amino acid. The "Red" color shows all polymorphic sites while the "underscores" represent conserve (>75%) amino acids. The "dashed lines" indicate indels, while the "Arrow" shows the C- terminal frameshift starting point.
